# Estimated time spent on preventive services by primary care physicians

**DOI:** 10.1186/1472-6963-8-245

**Published:** 2008-12-01

**Authors:** Kathryn I Pollak, Katrina M Krause, Kimberly SH Yarnall, Margaret Gradison, J Lloyd Michener, Truls Østbye

**Affiliations:** 1 Department of Community and Family Medicine, Duke University Medical Center; 2 Duke Comprehensive Cancer Center, Cancer Prevention, Detection and Control Research Program; 3 Duke NUS Graduate Medical School Singapore, 11 Hospital Drive, Level 4 Singapore 169610

## Abstract

**Background:**

Delivery of preventive health services in primary care is lacking. One of the main barriers is lack of time. We estimated the amount of time primary care physicians spend on important preventive health services.

**Methods:**

We analyzed a large dataset of primary care (family and internal medicine) visits using the National Ambulatory Medical Care Survey (2001–4); analyses were conducted 2007–8. Multiple linear regression was used to estimate the amount of time spent delivering each preventive service, controlling for demographic covariates.

**Results:**

Preventive visits were longer than chronic care visits (M = 22.4, SD = 11.8, M = 18.9, SD = 9.2, respectively). New patients required more time from physicians. Services on which physicians spent relatively more time were prostate specific antigen (PSA), cholesterol, Papanicolaou (Pap) smear, mammography, exercise counseling, and blood pressure. Physicians spent less time than recommended on two "A" rated ("good evidence") services, tobacco cessation and Pap smear (in preventive visits), and one "B" rated ("at least fair evidence") service, nutrition counseling. Physicians spent substantial time on two services that have an "I" rating ("inconclusive evidence of effectiveness"), PSA and exercise counseling.

**Conclusion:**

Even with limited time, physicians address many of the "A" rated services adequately. However, they may be spending less time than recommended for important services, especially smoking cessation, Pap smear, and nutrition counseling. Future research is needed to understand how physicians decide how to allocate their time to address preventive health.

## Background

Inadequate delivery of preventive health services is well documented [1]. Only 50% of smokers report receiving smoking cessation counseling beyond simple advice [2-4] and less than one-third of patients over 50 have had a blood stool test in the past two years [5].

Although there are numerous reasons for this lack of adequate care delivery, limited available time is one of the main barriers. If physicians were to provide all services recommended by preventive service guidelines, it has been estimated that it would require 7.4 working hours per day [6]. Because physicians clearly cannot spend this amount of time on prevention, they are forced to forego some services either by omitting them completely or addressing them only briefly. Uncertainty about how best to utilize the limited time spent with patients can be further compounded by multiple, competing, and often inconsistent guidelines regarding preventive health care, including most notably recommendations from the US Preventive Service Task Force (USPSTF) [7] and the American Cancer Society [8].

Inevitably, physicians must prioritize certain services over others. The USPSTF guidelines provide ratings for the level of evidence supporting each preventive service; physicians may also choose services they feel confident addressing or that they feel are most likely to result in patient behavior change or benefit [9-11]. Regardless of how physicians prioritize, preventive services still "compete" for time with acute problems and chronic care, and even with one another. For instance, when physicians document counseling more patients to quit smoking, their cancer screening rates decrease [12].

Few have documented the precise amount of time physicians spend on each preventive health service during preventive health and chronic care visits. Yawn and colleagues calculated the percent of time physicians spend discussing preventive health during chronic care visits, but did not translate percentages into actual minutes that physicians spend [13]. In that study, physicians spent over half their time taking histories and only 20% on health education. Further, they spent almost no time addressing nutrition (2.7% of total time), exercise (2.0% of time), or smoking (1.3% of time). The services physicians address can be affected by patient factors (e.g., patient motivation, health literacy), physician factors (e.g., knowledge of guidelines, outcome expectations that addressing behavior will improve patient health), and systems factors (e.g., reimbursement, time) [14,15]. In this report, we focus only on the systems factor of time as a constraint for the delivery of preventive services.

We examined National Ambulatory Medical Care Survey (NAMCS) data from 2001–2004 to estimate the time spent by primary care physicians (family physicians and general internists) when delivering important clinical preventive services. We compared these reports of actual time spent with the amounts of time recommended in the literature for the adequate delivery of each service. In this way, we move past the *rate *of preventive service delivery as a measure of adequacy to determine, when the services *are *delivered, whether they are delivered as recommended.

## Methods

The National Ambulatory Medical Care Survey (NAMCS) [16] documents the provision of ambulatory medical care services, regardless of specialty, in the United States. It is based on an annual national sample of visits to nonfederally-employed, office-based physicians primarily engaged in direct patient care. For each sampled visit, patient demographics, the time spent with the physician, and the diagnostic, preventive and therapeutic services provided during the visit are collected. Our analysis sample included data from the surveys conducted in 2001, 2002, 2003 and 2004 (combined n = 103,593). Analyses were conducted in 2007 and 2008.

The focus of our analysis was adult primary care; therefore, we included only visits to family physicians and general internists (n = 24,436). Physicians self-report the type of visit: preventive, chronic disease management, or acute care. Of these, we included preventive (n = 3,365) and chronic visits (routine and "flare-up"; n = 9,108) only, because we were interested in visits in which preventive services are typically provided; we modeled them separately, because the purpose and nature of the visits are different. The outcome variable for our analysis was time spent with physician, self-reported by the physician and recorded in minutes. If no physician was seen (e.g., a lab-only visit), the visit was not included in the analysis (n = 298 preventive; n = 430 chronic). Further, if a non-physician clinician was seen at the same visit, it might indicate team-based sharing of care, and affect the amount of time spent by the physician on services delivered. Therefore, we excluded a very small number of visits in which a physician assistant (n = 64 preventive; n = 100 chronic) or a nurse practitioner (n = 26 preventive; n = 65 chronic) was seen in addition to the physician.

Available patient variables included age (in years), sex, race (white, African American or other), ethnicity (Hispanic vs. not Hispanic), type of insurance (private, Medicaid, Medicare, or other), whether the patient was new or had been seen previously in the clinic, and tobacco use. Because many patients had missing information for ethnicity and tobacco use (and because there was no significant bivariate relationship between these variables and time spent with physician), these variables were not included in the multivariate analyses.

NAMCS includes data on different kinds of procedures or services provided during the visit according to certain categories: diagnostic/screening services (e.g., urinalysis, x-ray, Papanicolaou (Pap) test); counseling/education/therapy (e.g., asthma education, exercise, physiotherapy); surgical procedures (write-in), and medications/injections (write-in). As the focus of our study was preventive health services included in the USPSTF guidelines (specifically, the guidelines in place during the time of the study, 2001–2004) [8], we did not include the last two categories of services. Also, within the screening and counseling groups, we only included preventive services. The services documented in NAMCS and therefore included in our analyses were: 1) screening tests: blood pressure, cholesterol, Pap smear, colon scope (NAMCS terminology) procedures (i.e., colonoscopy and/or flexible sigmoidoscopy), mammography, and prostate specific antigen (PSA) test; and 2) counseling: tobacco cessation, diet/nutrition, and exercise. Physicians completed the NAMCS form by checking a box if they provided the service. When physicians checked the box, that could represent talking about the service (e.g., blood pressure) or actually providing the service (e.g., doing the Pap smear).

Because of the complex sampling design of the NAMCS survey, we used the SURVEYREG procedure in SAS (SAS 9.1, Cary NC, 2004) for our multivariate analysis. NAMCS utilized a multistage probability design that involved probability samples of primary sampling units (PSUs), physician practices within PSUs, and patient visits within practices [17]. The analyses met the criteria for cell frequencies and relative standard error suggested by NAMCS [18], indicating that the estimates were reliable. The dependent variable was the visit length in minutes.

In the multivariate model, an indicator for each specific service was used to represent whether or not that service was provided at that visit (0 = no, 1 = yes). In general linear modeling, the coefficient represents the increase in the dependent variable (time) for each unit increase in the independent variable (delivery of the service). Therefore, the coefficient of each service can be interpreted as the mean number of minutes that the visit length increased when the service was delivered, or more simply, the time spent when delivering the service. The multivariate model adjusted for demographic variables including age, sex, race, insurance type, and patient type (new vs. established). The study was declared exempt from review by the Institutional Review Board of the Duke University Medical Center.

## Results

Characteristics of the analysis sample are provided in Table 1, and the results of the multivariate analysis are presented in Table 2. The average preventive care visit was longer than the average chronic care visit (M = 22.4, SD = 11.8; M = 18.9, SD = 9.2 respectively). New patients, particularly during chronic care visits, seemed to have longer visits, as much as 6.8 minutes more. Services on which physicians spent relatively more time were PSA, cholesterol, Pap smear, mammography, exercise counseling, and blood pressure. Physicians spent more time per service during preventive care visits discussing cholesterol, exercise, and blood pressure than during chronic care visits. In contrast, the time spent delivering Pap smears and tobacco cessation counseling during chronic care visits was greater than when these services were delivered during preventive care visits.

**Table 1:** Sample characteristics and frequency of delivery of preventive services

	**Preventive**	**Chronic**
**Variable**	**Visits**	**Visits**
	**n = 2,977**	**n = 8,513**
	n (weighted %)	n (weighted %)
***Covariates***		
Age (mean, SD)	41.3 (23.5)	56.9 (19.0)
Gender		
Male	1272 (43.1)	3578 (42.2)
Female	1705 (56.9)	4935 (57.8)
Race		
White	2575 (85.7)	7385 (84.8)
Black	265 (9.2)	823 (11.6)
Other	137 (5.1)	305 (3.7)
Payment		
Private insurance	1778 (61.1)	4149 (49.3)
Medicare	449 (16.5)	2888 (35.4)
Medicaid	318 (9.3)	604 (6.2)
Other	432 (13.1)	872 (9.1)
New patient	444 (14.3)	325 (3.5)
		
Preventive services *		
Blood pressure	2222 (75.1)	7147 (84.2)
Cholesterol	727 (25.5)	1175 (14.8)
Pap smear	382 (11.8)	72 (0.77)
Colon scope	88 (2.7)	95 (1.3)
Mammogram	252 (8.0)	127 (1.3)
Prostate specific antigen	252 (8.8)	222 (2.6)
Tobacco cessation	163 (4.7)	324 (3.5)
Nutrition counseling	787 (25.8)	1958 (23.5)
Exercise counseling	579 (19.2)	1415 (16.4)

* Percentages sum to more than 100% because patient could have more than one service in each visit.

**Table 2:** Estimated minutes spent on preventive health services and effect of covariates on visit length, by type of visit; adjusted analyses (estimate (SE))*

**Predictor variable**	**Preventive Visits**		**Chronic Visits**		**USPSTF Rating^7^**	**Recommended Time^6^**
	Estimate	Standard Error	Estimate	Standard Error		
***Covariates***						
Age (an increase of 10 yrs)	0.02	(0.02)	0.04	(0.01)		
Gender (female)	-0.46	(0.70)	0.39	(0.25)		
Race						
Black	-1.19	(0.97)	0.60	(0.55)		
All Other	-1.00	(0.96)	0.33	(0.82)		
Payment						
Medicare	0.95	(1.04)	-0.33	(0.35)		
Medicaid	-0.08	(0.87)	0.92	(0.68)		
Other Types	-0.88	(1.14)	-0.98	(0.61)		
New patient	2.59	(0.85)	6.67	(0.95)		
						
***Preventive services***						
Blood pressure	2.54	(0.91)	1.45	(0.62)	A	0.25
Cholesterol	5.42	(1.15)	2.02	(0.64)	A	1.00
Pap smear	1.86	(0.83)	4.70	(1.57)	A	3.00
Colon scope	1.94	(1.88)	1.01	(1.11)	A	17.00
Tobacco cessation counseling	0.11	(1.20)	1.20	(0.63)	A	3.00
Mammography	2.54	(1.30)	2.87	(1.22)	B	1.00
Nutrition counseling	1.34	(0.68)	-0.17	(0.37)	B^†^	8.20
Prostate specific antigen	4.90	(1.27)	3.91	(0.97)	I	N/A
Exercise counseling	2.89	(0.92)	1.43	(0.48)	I	N/A

* Beta coefficients for the preventive services can be interpreted as the mean amount of time spent providing that service.

^† ^Nutrition is rated "B" only for those with documented related disorders (dyslipidemia or high BP).

### Estimated time spent versus time required for services

We compared the estimated time spent per service to estimates of time recommended to deliver each service adequately [6] (Table 2). The recommended times were derived from the literature and were conservative, assuming that in some cases, the only time recommended by physicians would be for ordering the test (i.e., cholesterol, mammography) or for glancing at a vital sign assessed by the nurse (i.e., blood pressure). According to the estimates from this analysis of the NAMCS data, the time spent delivering some "A" rated ("good evidence") services – blood pressure and cholesterol – met or exceeded the recommended time. Time spent on two other "A" rated services, tobacco cessation and Pap smears (in preventive visits), and one "B" rated ("at least fair evidence") service, nutrition, was less than what is recommended for adequate delivery of those services. It is notable that the time spent per service was relatively high for two services that have an "I" rating ("inconclusive evidence of effectiveness," e.g., *not *recommended) according to the USPSTF, namely PSA and exercise counseling.

## Discussion

There are several important findings from these analyses. First, the time spent on many important "A" and "B" rated preventive services, namely cholesterol, mammography, Pap smear (in chronic care visits), and blood pressure, met or exceeded the length of time that is recommended to deliver those services. One of the strongest predictors of a woman obtaining a mammogram is physician recommendation [19,20]. Primary care physicians seem to be spending time on these discussions, and by doing so, may be helping women detect breast cancer at earlier stages.

Our second finding is that the time spent on some other important "A" and "B" rated preventive services did not meet the recommendations for adequate delivery. These services were smoking cessation counseling, Pap smears, and nutrition counseling. In the case of tobacco cessation, for which only three minutes is the minimal time recommended, physicians spent at most 1.4 minutes during chronic care visits and virtually no time during preventive care visits. Physicians reported discussing smoking in only about 4% of all visits (Table 1), which is far below the national smoking rate of approximately 20% [21]; when physicians discussed smoking, visit time increased only minimally. Given evidence showing that smoking cessation counseling is one of the most effective and efficient ways physicians can promote prevention of mortality and morbidity [7,22,23], this potential lack of attention is concerning.

Our results confirm other work that shows that physicians do not address tobacco cessation consistently or adequately [24,25]. Physicians may not realize the impact they have when they counsel smokers to quit. Low self-efficacy to counsel smokers, low outcome expectancies that smokers will quit as a result of the counseling [26], poor systems for reminding them to counsel, and no time to address the issue adequately all also contribute to low cessation counseling rates. All factors, not just physician attitudes, must be addressed to increase tobacco cessation counseling [27].

Time spent delivering Pap smears during preventive visits did not meet recommendations, although this may be because it is a routine part of preventive care visits that may not need much explanation. In contrast, providing this service during chronic care visits appears to need more of a preamble as it is less obvious to the patient why it is brought up in the context of another disease. It is also possible that Pap smears addressed during chronic disease visits are for women who at higher risk for cervical cancer. That more time is spent on tobacco cessation during chronic care visits is not unexpected as smoking causes and/or compounds the negative effects of many common chronic illnesses including COPD and asthma, coronary heart disease, and peripheral and cerebrovascular disease [22,23].

A third important finding is that considerable time was spent delivering services that do not have conclusive evidence of efficacy and have received an "I" rating from the USPSTF. PSA screening, in particular, is a challenging topic because the various guidelines for prostate cancer screening are conflicting. The extra time spent may be devoted to explaining the complexity and inconsistent evidence for PSA testing – for example, the USPSTF has concluded there is insufficient evidence to recommend PSA screening in men under age 75 [28], while the ACS states that PSA should be explained to men aged 50 and older and that not offering or discouraging the test is "not appropriate" [8]. Further, physicians may talk about PSA tests out of fear of malpractice suits [29]. Exercise counseling is also somewhat complex. Given its impact on obesity prevention and control and known benefits to overall health, physicians may feel that discussing exercise is an essential part of an annual health check-up. This service has received an "I" rating, however, because evidence is inconclusive for the *efficacy *of exercise counseling to change behavior. Thus, physicians may be getting a mixed message from the guidelines about whether to attempt to help patients increase their physical activity.

Our analyses imply that physicians do not rely solely on the USPSTF guidelines to direct them in how to address preventive health. The well-noted lack of time to deliver the necessary services may cause physicians to spend less time discussing some important preventive services. With unsystematic guidance on which services to provide, biases in care can arise based on physicians' knowledge of guidelines, confidence level, and expectations about patient's response. Preventive service delivery would be improved if developers of guidelines worked together to develop and disseminate a single message to help physicians know how best to use their time. The USPSTF guidelines begin to serve this purpose, but multiple independent guidelines from a number of other organizations are also available (e.g., American Cancer Society, Joint National Committee on Prevention, Detection, Evaluation, and Treatment of High Blood Pressure [30], etc.). Very recently, the Agency for Healthcare Research and Quality (AHRQ) released a tool called the "Electronic Preventive Services Selector" to help clinicians make decisions about which services to provide [31].

In addition to better guidelines, a possible solution to the lack of primary care physician time is to use team-based approaches in which physicians are not responsible for delivering all preventive health services [32,33]. Nurses and health educators can address more of the counseling, while physicians handle the more complicated cases of chronic care disease management [34]. While there are some risks involved in the team-based approach, including a patient perception of fragmented or poorly-coordinated care [35], team-based care as posited in Wagner's Chronic Care model should provide better quality of care and patient satisfaction than the traditional solo physician model [36,37].

### Limitations and strengths

The NAMCS did not collect data on all "A" and "B" rated preventive health services; therefore, a complete comparison between what is recommended and what is delivered was not possible. Also, some have reported that NAMCS data may underestimate behavioral counseling and overestimate time spent when compared to direct observation [38]. This should be less of a problem as our method compared the difference between visits with and without the service provided rather than a report of the time spent on each service. However, we acknowledge that our calculations are estimates rather than actual observed measurements. Finally, our analyses do not address whether a service was due for the patients who received them, only the estimated amount of time spent when the service was delivered. Thus, a physician may have discussed mammography with a woman who was not due for a mammogram but still checked the box "mammogram". Whether the service was due is not relevant for this report, however, as we are attempting to describe the time spent when delivering a preventive service, not *whether *physicians are delivering preventive services to those who require them. We also cannot determine if a service was delivered in another visit by a separate care provider (e.g., a physician assistant or nurse practitioner in a team-based model); however, if it was, it is likely that the physician would not have addressed the service at all, and therefore the mean time used when the service *was *delivered would not be affected.

## Conclusion

Using a national data set to investigate the actual time spent in the delivery of specific clinical preventive services, our analyses support the notion that too little time is spent on prevention. With already large and rapidly growing competing demands and guidelines, physicians may rely on their personal intuition or bias, office systems reminders, chronic illness triggers [39], or patients' requests to determine the time they will spend on preventive services during each visit. The solution is not for physicians to do more. Indeed, with the current physician shortage [40], physicians will likely be able to do less. One solution is to help physicians prioritize preventive care that *they *themselves can provide best and to delegate services that other members of the health care team can provide. Otherwise, the result will be more missed preventive care delivery and more need for tertiary care treatment.

## Competing interests

The authors declare that they have no competing interests.

## Authors' contributions

KP conceived of and designed the study and drafted the manuscript. KK participated in the design of the study, performed the statistical analysis and edited the manuscript. KY, MG and JLM participated in the study design and edited the manuscript. TØ participated in the study conception, design and data analysis and helped draft the manuscript. All authors read and approved the final manuscript.

## Pre-publication history

The pre-publication history for this paper can be accessed here:


